# Effects of Different Drying Methods on the Quality of *Amomum villosum* Lour. Based on GC-MS and Chemometric Techniques

**DOI:** 10.3390/foods15081404

**Published:** 2026-04-17

**Authors:** Zhaoyou Deng, Jing Yu, Cuiyun Yin, Yin Yuan, Deying Tang, Shifang Liu, Xuanchao Shi, Lixia Zhang, Yihang Li

**Affiliations:** 1 Yunnan Branch of Institute of Medicinal Plant Development, Chinese Academy of Medical Sciences & Peking Union Medical College, Jinghong 666100, China; pharmacologyvip@163.com (Z.D.); yujingynu1113@163.com (J.Y.); cyyin@implad.ac.cn (C.Y.); swyuanyin@126.com (Y.Y.); tdy629@126.com (D.T.);; 2 Yunnan Key Laboratory of Southern Medicine Utilization, Yunnan Branch of Institute of Medicinal Plant Development, Chinese Academy of Medical Sciences & Peking Union Medical College, Jinghong 666100, China

**Keywords:** *Amomum villosum* Lour, GC-MS, drying process, essential oil, bornyl acetate

## Abstract

Postharvest processing plays an important role in improving the quality and storage stability of mature fresh *Amomum villosum* Lour. (*A. villosum*). We investigated the effects of seven common drying methods (electric baking drying (EBD), heat pump drying, sun drying after heat pump drying, shade drying, hot air drying, sun drying, and freeze drying) on the volatile components of Amomum villosum. To discriminate different samples and identify key markers, chemometric techniques, including principal component analysis (PCA) and orthogonal partial least squares discriminant analysis (OPLS-DA), were applied to Chromatography–Mass Spectrometer (GC-MS) data of 70 identified metabolites. As an unsupervised method, PCA was first utilized to observe the overall clustering tendency of 21 samples, showing clear dispersion among seven groups with a slight overlap in the samples from sun drying after heat pump drying and hot air drying. To improve discrimination accuracy, the OPLS-DA model was further established as a supervised method. Its reliability was verified by permutation tests and cross-validation, which confirmed the absence of overfitting (R^2^ and Q^2^ intercepts with the vertical axis were <1 and <0, respectively). S-plots combined with variable importance in projection (VIP) values greater than 1 were used to screen differential metabolites, and camphor, borneol, and bornyl acetate were identified as the key discriminant markers for the samples obtained by different drying methods. Consequently, camphor, borneol and bornyl acetate, which are regarded as quality markers of *A. villosum*, were determined by gas chromatography (GC) to identify the optimal drying method for fresh *A. villosum*. The results showed that the content of the quality markers in *A. villosum* obtained by the seven drying methods outclass the standards of the Chinese Pharmacopoeia.Comprehensively considering the experimental results and the convenience and operability of the drying process, EBD is the most suitable drying process of *A. villosum* for popularization and application. It is on account of the shortest drying time among the seven drying methods, which only took 21.63 h to complete the drying of fresh *A. villosum*. Besides that, the quality control parameters in the content of bornyl acetate, camphor, borneol and the essential oil of *A. villosum* obtained by EBD were far more than the standards stipulated in the pharmacopeia.

## 1. Introduction

*A. villosum* was a perennial herb of dual-purpose for medicine and food, with a long medical history of more than one thousand years for treating gastrointestinal diseases, pregnancy-related vomiting and preventing miscarriages in China [1,2,3,4]. *A. villosum* is mainly distributed in southern and southwestern areas of China and Southeast Asian countries, such as Laos and Vietnam, which are located in the tropical and subtropical monsoon climates [5]. A considerable number of modern pharmacological studies have demonstrated that *A. villosum* possesses multiple bioactivities, such as antioxidant, anti-tumor, anti-inflammatory, anti-diarrhea, anti-ulceration, and bacteriostasis activities [6,7,8,9]. The main chemical components in *A. villosum* are essential oils, saponins, flavonoids, terpenoids, phenols, and polysaccharides [10,11,12]. To date, its essential oils may be the main active ingredient and one of the most important quality criteria of *A. villosum* [13]; the most common components are bornyl acetate, camphor, α-cadinol, borneol, linalool, β-myrcene, D-limonene, and terpinolene [14,15]. Notably, essential oils in *A. villosum* are volatile and thermosensitive, and their composition, content, and bioactivity are highly susceptible to drying processes, which is a key factor affecting the final quality of *A. villosum* products [16]. Drying temperature, drying rate, and drying time, which vary among different drying methods, can directly induce changes in the essential oil components of herbs [17]. For instance, high-temperature drying methods (such as hot air impingement drying) may cause thermal degradation or volatilization of heat-labile essential oil components, leading to a significant reduction in the content of key active compounds, especially bornyl acetate [18]. Bornyl acetate, as the core active component in *A. villosum* essential oil, is closely related to its medicinal efficacy (such as regulating gastrointestinal function and relieving vomiting), and its content stability is crucial for ensuring the clinical effect and commercial value of *A. villosum* [19]. In contrast, low-temperature drying methods (such as freeze-drying) may better retain the original composition and content of essential oils, including bornyl acetate, but their application is often limited by high energy consumption and complex operation [20,21]. Meanwhile, the content and activity of the essential oil and bornyl acetate are considered to be extremely vital indicators of the quality of *A. villosum* in the 2020 version of the Chinese Pharmacopoeia [22]. Therefore, clarifying the effects of different drying methods on the essential oil composition, especially the content and stability of bornyl acetate, is of great significance for optimizing the drying process of *A. villosum*.

The mature fresh fruit of *A. villosum* is a seasonal plant with a very short storage life; unsuitable storage may cause mold, which would influence the nutritional composition, efficacy and quality of *A. villosum* [23]. Therefore, drying is currently the main method to improve the availability of *A. villosum* fruit products, ensuring their medicinal and nutritional value. The quality of *A. villosum*, especially in the content and chemical constituents of essential oils, may be influenced by various drying methods, such as freeze-drying (FD), hot air drying (HAD), shade drying (SHD), sun drying (SD), microwave drying, electric baking drying, heat pump drying (HPD) and smoke drying [24,25,26]. SD and SHD are highly weather-dependent, and dried herbs easily reabsorb moisture under high humidity or sudden rain. Although both methods employ low temperatures that may help preserve heat-sensitive volatile oils, their slow drying rates prolong post-harvest metabolism, leading to the loss of volatile components and reducing herb quality [27]. FD effectively preserves essential oils and bioactive components by minimizing degradation under low-temperature vacuum conditions, yet its high energy consumption, slow rate and high cost restrict its widespread use [28]. HAD is efficient and low-cost, but its high temperature easily causes volatilization, oxidation and thermal degradation of volatile oils, resulting in significant loss of key components [29]. HPD can effectively induce the formation of a crust layer on the surface of partially dried materials, which serves as a protective barrier and reduces the diffusion and loss of high-molecular-weight volatiles [29]. EBD is a controllable thermal processing method that converts electricity to uniform heat via resistance heating and forced air convection. It enables accurate temperature–time control, good reproducibility, and reduced operator-dependent variation, helping preserve active ingredients in herbal medicines [24,30].

Given the respective advantages and limitations of these drying methods, it is vital to find a suitable drying technology to achieve the desired quality of *A. villosum* fruit products under relatively low energy consumption and easy popularization for application. In addition, to the best of our knowledge, less attention has been paid to the choice of *Amomum villosum* fruit on drying methods based on systematically evaluating drying characteristics, convenience, and the quality of *A. villosum*. Therefore, this study adopted the GC-MS technique combined with chemometric techniques to investigate the effect of seven drying method on volatile oil components in *A. villosum* for identifying quality markers; the contents of these quality markers were then determined by GC-FID to compare the different drying methods effects on the quality of *A. villosum,* aiming to explore a scientific, reasonable, convenient and quick drying method for the drying treatment of fresh *A. villosum*.

## 2. Materials and Methods

### 2.1. Plant Materials

Approximately 60 kg of mature fruits of *A. villosum* were collected in August 2023 from Jingha Hani Township (Xishuangbanna Dai Autonomous Prefecture, Jinghong, Yunnan Province, China). All the samples were identified as the fruit of *A. villosum* Lour. of Zingiberaceae by Dr. Liuxia Zhang (Yunnan Branch Institute of Medicinal Plant, Chinese Academy of Medical Sciences, Jinghong, China). The moisture content of the fruit was immediately measured on arrival, which was determined using the oven method (105 °C for 24 h) in triplicate. A total of 100 g of *A. villosum* was randomly selected and baked to a constant weight. The moisture content of *A. villosum* was calculated by the ratio of reduced weight to initial weight, and the initial moisture content of fresh *A. villosum* was 74.73% on a wet basis. The standard chemicals borneol, camphor, and bornyl acetate were obtained from the National Institutes for Food and Drug Control of China.

### 2.2. Drying Method

Heat pump drying (HPD): a total of 1.5 kg of *A. villosum* fruit was dispersed uniformly on a stainless mesh tray and dried in a heat pump drying oven (CT-C-0, Nanjing Focus Machinery Equipment Co., Ltd., Nangjing, China) at a temperature of 60 °C and a humidity of 5% for 28.95 h, which ensured the moisture of the material to be less than 15%, packed in a clean self-sealing bag, and stored at room temperature until use; the same below.

After heat pump drying and sun drying (HPD-SD): a total of 1.5 kg of *A. villosum* fruit was dispersed uniformly on a stainless mesh tray and dried in a heat pump drying oven (CT-C-0, Nanjing Focus Machinery Equipment Co., Ltd., Nangjing, China) at a temperature of 60 °C and a humidity of 5% for 10 h, and then dried under the sun at a temperature of 22~28 °C for 14.78 h.

Freeze-drying (FD): *A. villosum* fruit (0.5 kg) was laid on a pallet and dried in a freeze-dryer (FD5-3, SIM, Gold Sim (Beijing) Instruments Co., Ltd., Beijing, China) at a temperature of −50 °C and a vacuum of 13.33 Pa for 22 h.

Hot air drying (HAD): a total of 1.5 kg of *A. villosum* fruit was dispersed uniformly on a stainless mesh tray and dried in a hot air drying oven (FCD-3000 Seriab, Beijing Ever Bright Medical Treatment Instrument Co., Ltd., Beijing China) at a temperature of 60 °C for 61.72 h.

Electric baking drying (EBD): referring to the method used by Mei et al. [31], some parameters have been modified. A total of 1.5 kg of *A. villosum* fruit was dispersed uniformly on a stainless mesh tray and dried in a box-type high-efficiency electric energy dryer (SKHG-1MDZAG, Changzhou Punaier Drying Equipment Co., Ltd., Changzhou, China) at a temperature of 60 °C for 21.63 h.

Sun drying (SD): fresh *A. villosum* fruit (1.5 kg) was dispersed uniformly on a stainless mesh tray and dried under direct sunlight at a temperature of 22~28 °C for 54.97 h. The fruits were placed in a cool and ventilated place overnight for natural air-drying without any other treatment, and only the duration of sunlight exposure was recorded.

Shade drying (SHD): a total of 1.5 kg of *A. villosum* fruit was dispersed uniformly on a stainless mesh tray and dried under local natural conditions in a room for 816.00 h.

### 2.3. Qualitative Profiling of Essential Oil of A. villosum by GC-MS

#### 2.3.1. Essential Oil Preparation

The essential oil of *A. villosum* was obtained by steam distillation, according to a method described in the Chinese Pharmacopeia (2020 edition), as follows: First, the dried samples of *A. villosum* were powdered and filtered through a 30-mesh sieve. Then, approximately 30.0 g powder of *A. villosum* was weighed and placed in a 1000 mL round-bottom flask with 500 mL H_2_O; the flask was heated in an electric heating sleeve until boiling and kept slightly boiling for 5 h and left for 1 h to collect the essential oil of *A. villosum* [32]. The oil yields were calculated via the volume of the essential oil per 100 g of *A. villosum*. Subsequently, 1 mL of the essential oil was diluted to 100 mL using chromatographic-grade n-hexane. Finally, the obtained compound was filtered through a 0.22 μm filter head and then stored in sealed brown vials at 4 °C for GC-MS analysis.

#### 2.3.2. GC-MS Analysis

The GC-MS instrument was an Agilent Technologies 7890A gas chromatograph (Agilent Technologies, Santa Clara, CA, USA) combined with a 5975C MSD mass spectrometer. For manual data evaluation, the MassHunter (Version B.08.00) software was used. The GC conditions were as follows: split injection mode with a split ratio of 10:1, HP-5MS column (30 m × 0.25 mm i.d. × 0.25 μm film thickness), injection port temperature 270 °C, interface temperature 220 °C, and helium carrier gas flow 1 mL/min; the column temperature programmed ramp-up start at 60 °C, hold for 1 min, ramp up to 115 °C at 3 °C/min, hold for 1 min, ramp up to 160 °C at 1.7 °C/min, ramp up to 230 °C at 10 °C/min, and post-run at 230 °C for 5 min. The MS conditions were as follows: full scan mode, ionization energy 70 eV, ion source temperature 230 °C, quadrupole temperature 150 °C, the mass scan range (*m*/*z*) was 40–500 amu with a scanning speed of 1.5 scan/s, and the solvent delay time was 2.00 min. The compounds were identified by comparing the mass spectra with the NIST 20 library, and only those with a matching degree higher than 90% were selected. The identification results were further verified and confirmed by referring to the relevant published literature [10,14,21,22,33]. The relative mass percentage of each component was determined using the area normalization method.

### 2.4. Quantitative Analysis of A. villosum by GC-FID

#### 2.4.1. Preparation of Camphor, Borneol, and Bornyl Acetate of *A. villosum* for GC Analysis

The powdered (1.0 g, 50 mesh) seed mass of *A. villosum* was weighed, 25 mL of absolute ethanol was added, sonicated (300 W, 40 kHz) for 30 min, cooled to room temperature, and the absolute ethanol was used to make up the lost weight, filtered through a 0.22 μm filter head, and stored in sealed brown vials at 4 °C until use, which was the test substance solution. The calibration curves for calculating the content of camphor, borneol and bornyl acetate in *A. villosum* were drawn via the series concentrations.

#### 2.4.2. GC-FID Instrumentation

The measurement was carried out using a GC system (Agilent GC 7890A, Agilent Technologies, Santa Clara, CA, USA) equipped with a hydrogen flame ionization detector. The GC operating conditions were as follows: The GC column was operated at 100 °C with a DB-1 capillary column (30 m × 0.25 mm × 0.25 μm). The temperature of the inlet and detector was 230 °C and 250 °C, respectively. Split injection was performed at a split ratio of 10:1.

### 2.5. Data Processing and Analysis

All samples were measured three times in parallel, and to ensure reproducibility of the data, compounds that appeared in at least two parallel trials were used as the analytes. The data obtained were analyzed qualitatively using the NIST Chemical Structures library (2020). Chemometric analyses (including PCA and OPLS-DA) were performed using SIMCA 14.1 software. All data were subjected to mean centering and unit variance scaling before analysis. Origin 2024 and Photoshop were used for plotting, data processing, and statistical analysis.

## 3. Results

### 3.1. Analysis of the Chemical Composition in the Essential Oil of A. villosumin Obtained by Different Drying Methods

In the present research, a total of 70 volatile components, with 49 compounds in common, were identified by GC-MS in the essential oil of *A. villosum* from seven drying methods, and the relative contents were obtained by their peak area ratios. And these data are shown in Table 1. Among those compounds, bornyl acetate, borneol, camphene and camphor were confirmed to be the main constituents of *A. villosum*, whose content accounted for more than 5% of all the volatiles. Camphor, borneol and bornyl acetate were the components with higher contents in the essential oil, and the relative content of bornyl acetate was the highest among all components.

### 3.2. Modeling and Model Evaluation of the Volatile Component of A. villosum Obtained by Different Drying Methods

**Figure 1:**
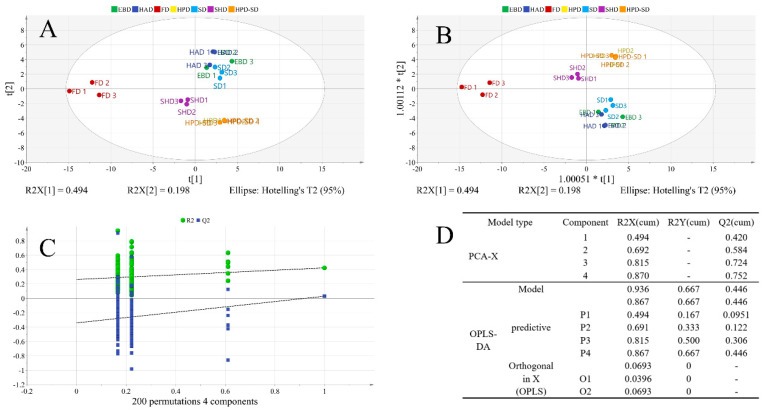
Score plot of the PCA-X model (**A**), OPLS-DA model (**B**), cross-validation of the OPLS-DA model (**C**), and model parameters of the volatile component of *A. villosum* obtained by seven drying methods (**D**).

To provide more information about the differentiation of the samples obtained by the seven different drying methods, PCA was performed based on the relative content of 70 components in Table 1. As shown in Figure 1A,B, the sample of *A. villosum* obtained by the seven drying methods was within the 95% confidence interval. In the PCA-X model, seven groups of samples were dispersed; the samples of HPD-SD and HAD had some overlap, but there was no influence to exactly distinguish them, while the clustering of FD samples was unsatisfactory. In the OPLS-DA model, all the samples in the seven groups were clustered preferably; in addition, the samples of HPD and HPD-SD overlapped, and the other samples were well distinguished. In order to prevent a possible overfitting of the OPLS-DA model, which could weaken its ability to assess the new sample dataset to a certain extent, the model’s reliability was verified via the permutation test and cross-validation analysis. As shown in Figure 1C, the OPLS-DA model had no overfitting, which indicates that the model is stable and reliable. The intercepts of the R2 and Q2 curves with vertical coordinates were lower than 1, and the intercept of Q2 on the vertical coordinates was below 0. Furthermore, the significant probability *p* value was lower than 0.05 in the CV-ANOVA analysis. It can be seen in Figure 1D that the OPLS-DA model featured values R2X = 0.936 and Q2 = 0.446, and showed more data deviation than the PCA-X model with R2X = 0.870 and Q2 = 0.765. Hence, the OPLS-DA model could preferably distinguish the samples of *A. villosum* obtained by the seven drying methods under study compared to the PCA-X model.

### 3.3. Excavation of Potential Differences in Volatile Components of A. villosum Obtained by Seven Different Drying Methods

**Figure 2:**
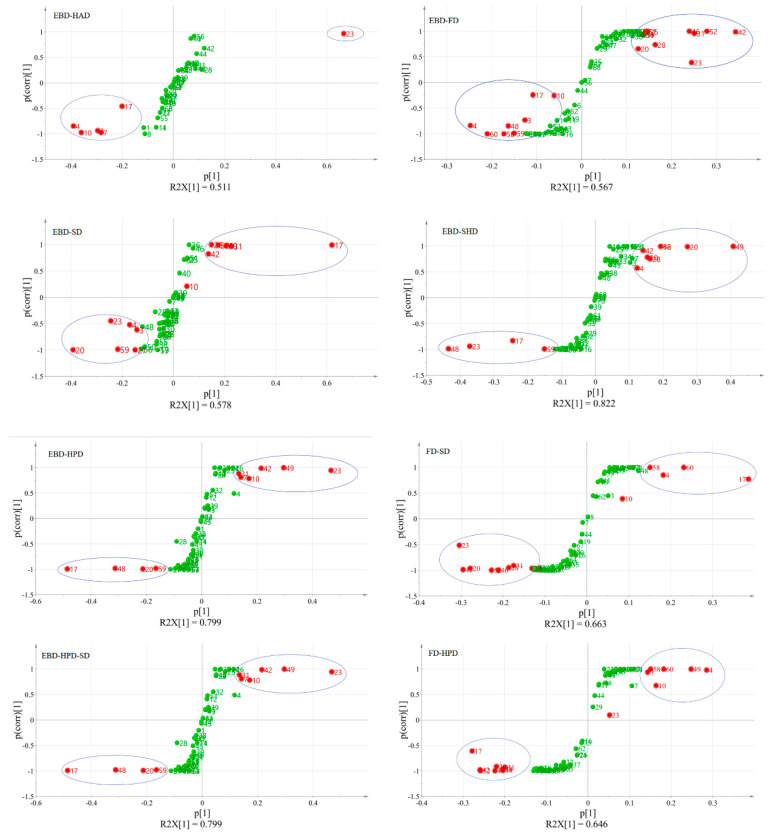
S−plot of volatile component-based OPLS-DA model analysis of *A. villosum* samples obtained via seven drying methods.

S-plots were applied to confirm chemical composition differences between the two samples and helped to identify metabolites of statistical and potential biochemical significance. The points at the ends of the S-plot demonstrated variables with the highest contributions to the model, while those with smaller contributions were clustered near the origin. The OPLS-DA model demonstrated that the *A. villosum* in samples obtained by the other six drying methods were better separated from the EBD samples. Given this, the study pays close attention to breaking down the differences in volatile components. Under the above conditions, the S-plot of volatile components is shown in Figure 2. The red dots in Figure 2 indicate metabolites with VIP > 1. Furthermore, the three components that differed most significantly in comparison between EBD and the other six samples were camphor, borneol, and bornyl acetate. In the comparison of EBD with HPD and HPD-SD samples, they have the same results, and the components with the most significant differences in the volatile component (VIP > 2) were camphor, borneol, and bornyl acetate. In the comparison of FD with HPD and SD samples, the same volatile components with VIP > 2 were camphor, borneol, and bornyl acetate. Based on the above analysis, it can be drawn that camphor, borneol, and bornyl acetate were the most affected components in different drying processes. Changing the train of thought, the changes in the content of camphor, borneol, and bornyl acetate can be used to confirm the optimum drying method of fresh *A. villosum*.

### 3.4. Comparison of the Contents of Essential Oil, Camphor, Borneol and Bornyl Acetates of A. villosum Obtained by Different Drying Methods

**Figure 3:**
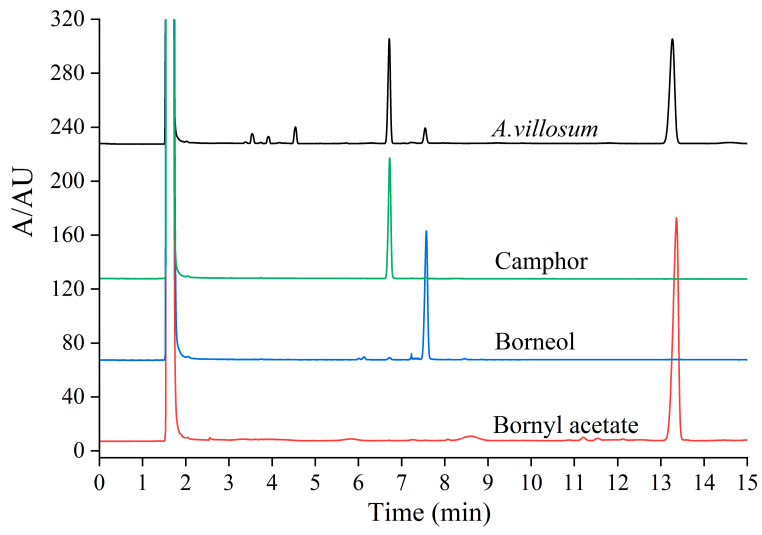
The gas chromatogram for chromatographic peak identification of *A. villosum.*

**Figure 4:**
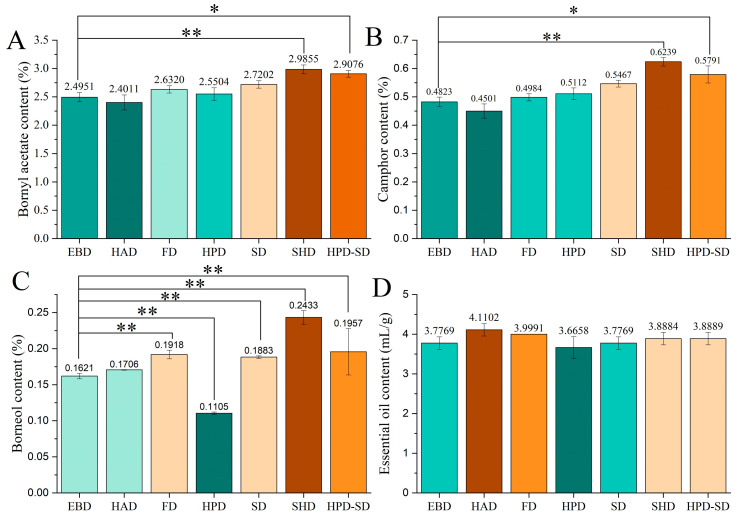
Comparison of the contents of bornyl acetates (**A**), camphor (**B**), borneol (**C**) and essential oil (**D**) of *A. villosum* obtained by seven drying methods. Data are shown as means ± SD. The significant differences were calculated by the Student’s *t*-test and expressed as an asterisk (* represents *p* < 0.05, ** represents *p* < 0.01).

As shown in Supplementary Figure S1 and Figure 3, the camphor, borneol and bornyl acetate peaks were well separated under the given GC operating conditions in Section 2.4.2. The respective regression equations of camphor, borneol and bornyl acetate shown in Supplementary Figure S2 were as follows: y = 2885.5x − 11.266 (R^2^ = 0.9999); y = 1418.9x − 2.2353 (R^2^ = 0.9999); and y = 1154.5x − 1.1458 (R^2^ = 0.9999). They showed a good linear relationship in the range of 0.47 mg/mL to 0.750 mg/mL, 0.025 mg/mL to 0.816 mg/mL, and 0.08 mg/mL to 0.960 mg/mL, respectively. The content of camphor, borneol, and bornyl acetate was calculated as average ± standard deviation (SD) (*n* = 3) via the respective regression equations. It can also be observed from Figure 4 that the content of camphor, borneol and bornyl acetate in *A. villosum* obtained by the drying method of SHD, HPD-SD, and SD was higher than the other drying methods with long-time high-temperature baking. There is an interesting phenomenon, which was the contents of essential oil (not less than 3.0%) and bornyl acetates (not less than 0.9%) in *A. villosum* obtained by the seven drying methods were much higher than the standards of the Chinese Pharmacopoeia. Considering the experimental results and the drying time (shown in Supplementary Figure S3), energy consumption, and operability of the drying process, EBD is the most suitable drying process for *A. villosum* for popularization and application. Its drying time is the shortest among the seven drying methods; only 21.63 h is required to complete the drying of fresh *A. villosum*, and the quality control parameters with bornyl acetate and essential oil content of *A. villosum* obtained by EBD are far more than the standards stipulated in the pharmacopoeia.

## 4. Discussion

Drying methods significantly influenced the release or retention of essential compounds [34]. The main essential components of *A. villosum* samples were α-pinene, camphene, β-myrcene, limonene, camphor, bornyl acetate, α-copaene, β-Elemene, and 3,6,6,9-tetramethyl-1,4,4a,5,7,9a-hexahydrobenzo [6]annulene, and consistent with the earlier results of Tu et al. [21]. Camphor, borneol, and bornyl acetate, among the volatile components in *A. villosum* samples, are easily influenced by the drying processes, probably because bornyl acetate, borneol and camphor were the three highest percentage compounds and accounted for more than half of all oil samples. It is confirmed that the volatile components in *A. villosum* were dominated by bornyl acetate, camphor and borneol, which were consistent with the previous studies [33,35]. The content of bornyl acetate and essential oil as quality markers of *A. villosum* were obtained by seven drying methods, which met the relative regulations in the Chinese Pharmacopoeia [36]. In addition, the contents of bornyl acetate and camphor in *A. villosum* obtained by FD, HPD-SD, SD, and SHD were significantly higher than those in heat-treated EBD, HPD, and HAD. This could be attributed to the high temperature in thermal drying processes, which accelerates the degradation and volatilization of thermally unstable volatile components [37,38]. No significant difference was observed in the total essential oil content of *A. villosum* among all drying treatments. This may be attributed to the relatively low drying temperature (60 °C), which reduced the volatilization loss of essential oil [18]. Therefore, we can affirm the optimum drying method for mature fresh *A. villosum* by comparing the convenience, drying time, and the difficulty level of popularization for different drying methods. FD is attributed to the low operating temperature and the preservation of the complete tissue structure of the sample [39]. Nevertheless, the high energy consumption and low production efficiency limit its utilization, and the freeze-drying process has main applications in nanoparticulate systems, microorganisms, genes, and vaccine formulations [40,41]. SHD and SD are low-energy consumption and environmentally friendly drying methods, but they are easily affected by the weather and have some shortcomings, including long drying time, serious separation of shell and seed mass, poor aroma and going moldy in storage [42,43]. In addition, the harvest season of *A. villosum* was mainly during the rainy season in the local climate. The humid and rainy environment is an unfavorable factor for the drying and storage of fresh amomum, as it causes mildew, poor quality and loss of use value [44,45]. Although HAD is the method with the lower drying rate and the poor quality of the product, convenience and low cost make hot air drying a common process for drying the medicinal herbs [46,47]. However, the drying time of HAD was 61.72 h (shown in Supplementary Figure S3), which was significantly longer than that of EBD. This may be attributed to the lower dewatering capacity of the HAD equipment compared with EBD. HPD is the method that is highly efficient and energy-saving and utilizes the principles of the inverse Carnot cycle, which is widely applied in food drying due to its low energy consumption, precise temperature control, and broad applicability [48,49]. EBD using an electric baking oven with a gas exchange system has the advantages of discharging the moisture generated by the drying processes, which offers high heat transfer rates due to the thin boundary layer formed on the surface of the material by high-velocity hot air impinged from the nozzles. Furthermore, HPD and EBD were the drying methods widely used in folk because these drying methods can dry more samples with lower energy consumption and high efficiency. Compared to HPD, EBD may be a more suitable drying method for postharvest processing of *A. villosum*. Although there was no obvious difference in the content of bornyl acetate, camphor and essential oil of *A. villosum* obtained by heat pump drying and electric baking drying, the content of borneol in *A. villosum* obtained by electric baking drying was superior to that of heat pump drying.

Taken together, the above results clearly demonstrate that drying techniques not only affect the content of individual volatile components but also determine the processing adaptability and economic cost of post-harvest treatment for *A. villosum*. Considering the comprehensive performance in component retention, drying time, energy consumption, and suitability for local rainy climatic conditions, EBD with the shortest drying time exhibited obvious advantages over other drying methods in this study, because drying time is directly related to energy consumption and the retention of volatile compounds, which is of great importance in mechanized post-harvest processing. It not only satisfied the quality requirements specified in the Chinese Pharmacopoeia but also achieved higher borneol content and better overall flavor and medicinal quality compared with HPD. Meanwhile, this method avoids the limitations of long drying times, weather dependence, and microbial contamination associated with natural drying, as well as the high cost and low efficiency of freeze drying. Therefore, electric baking drying can be recommended as a practical, efficient, and economically feasible drying strategy for the large-scale post-harvest processing of fresh mature *A. villosum*, which is conducive to ensuring the stable quality and commercial value of this medicinal material in actual production.

## 5. Conclusions

This study evaluated the effects of seven drying methods on the volatile components of fresh *A. villosum*. GC-MS combined with PCA and OPLS-DA was used to screen key quality markers. A total of 70 volatile components were identified; bornyl acetate, camphor, and borneol were confirmed as core differential markers (VIP > 1) and main quality markers of A. villosum, whose relative content accounts for over half of the total essential oil. GC-FID quantitative analysis showed that essential oil and bornyl acetate contents in all dried samples exceeded Chinese Pharmacopoeia standards.

Considering quality, drying time, energy consumption, and operability, EBD was the most suitable for popularization. It had the shortest drying time (21.63 h, shown in Supplementary Figure S3), favorable contents of key quality indicators, including bornyl acetate, camphor, borneol and total essential oil, and exhibited the advantages of simple operation and low energy consumption, simple operation, and low energy consumption.

## Figures and Tables

**Table 1:** Composition of the relative contents of the essential oil of *A. villosum* obtained via seven drying methods.

No.	Retention Time	CAS	Component	Relative Content (%)						
				EBD	HAD	FD	HPD	SD	SHD	HPD-SD
1	4.758	508-32-7	Tricyclene	0.18	0.17	0.15	0.17	0.17	0.18	0.17
2	4.859	2867-05-2	3-Thujene	0.05	0.05	0.05	0.05	0.05	0.05	0.05
3	5.027	80-56-8	α-Pinene	1.44	1.36	1.31	1.44	1.35	1.51	1.44
4	5.408	79-92-5	Camphene	6.94	6.80	6.51	7.01	6.77	7.02	7.01
5	5.547	24254-55-5	2-Hydroperoxyhexane	0.06	0.06	-	-	0.05	0.06	-
6	6.127	18172-67-3	β-Pinene	0.06	0.06	0.06	0.07	0.06	0.06	0.07
7	6.529	123-35-3	β-Myrcene	3.26	3.18	3.23	3.35	3.25	3.32	3.35
8	6.920	99-83-2	α-Phellandrene	0.36	0.35	0.35	0.36	0.35	0.37	0.36
9	7.302	99-86-5	α-Terpinene	0.05	0.05	-	0.05	0.05	0.03	0.05
10	7.723	5989-27-5	Limonene	7.32	7.19	7.18	7.46	7.35	7.44	7.46
11	7.793	470-82-6	Eucalyptol	0.13	0.12	0.11	0.12	0.12	0.12	0.12
12	8.715	99-85-4	γ-Terpinene	0.10	0.10	0.06	0.10	0.10	0.08	0.10
13	9.031	15537-55-0	cis-4-thujanol	-	-	0.07	-	-	0.05	-
14	9.778	586-62-9	Terpinolene	0.20	0.19	0.17	0.19	0.20	0.19	0.19
15	10.168	17699-16-0	4-Thujanol	-	-	0.06	-	-	0.04	-
16	10.251	78-70-6	Linalool	0.10	0.10	0.09	0.09	0.10	0.09	0.09
17	12.063	464-49-3	Camphor	22.68	22.52	22.21	21.54	23.70	22.19	21.54
18	12.119	465-31-6	Camphene hydrate	0.16	0.15	0.12	0.14	0.15	0.13	0.14
19	12.412	124-76-5	DL-Isoborneol	0.18	0.18	0.17	0.18	0.17	0.19	0.18
20	12.811	507-70-0	Borneol	3.53	3.50	3.64	3.32	3.11	3.96	3.32
21	13.261	562-74-3	Terpinen-4-ol	0.19	0.18	0.10	0.18	0.19	0.13	0.18
22	13.825	98-55-5	α-Terpineol	0.08	0.07	0.05	0.06	0.07	0.05	0.06
23	18.021	76-49-3	Bornyl acetate	39.70	40.20	40.38	40.80	39.27	38.98	40.80
24	19.495	3242-08-8	1-ethenyl-1-methyl-4-propan-2-ylidene-2-prop-1-en-2-ylcyclohexane	-	-	0.05	0.04	-	-	0.04
25	19.913	29873-99-2	(1R,2R)-1-ethenyl-1-methyl-4-propan-2-ylidene-2-prop-1-en-2-ylcyclohexane	0.23	0.23	0.36	0.27	0.25	0.25	0.27
26	20.506	14912-44-8	Ylangene	-	-	0.07	0.06	0.06	0.07	0.06
27	20.520	50894-66-1	α-Funebrene	0.06	0.06	-	-	-	-	-
28	21.692	3856-25-5	α-Copaene	1.94	2.01	2.17	1.89	1.92	2.16	1.89
29	22.161	33880-83-0	Elemene	0.10	0.10	0.11	0.11	0.10	0.10	0.11
30	22.400	54324-03-7	Bicyclosesquiphellandrene	0.10	0.10	0.19	0.10	0.10	0.13	0.10
31	22.524	515-13-9	β-Elemene	1.92	1.92	2.28	2.02	2.06	1.93	2.02
32	23.761	87-44-5	Caryophyllene	0.26	0.27	0.30	0.28	0.26	0.28	0.28
33	23.895	512-61-8	α-Santalene	0.49	0.50	0.59	0.49	0.48	0.53	0.49
34	24.719	18252-46-5	α-cis-bergamotene	0.56	0.57	0.67	0.57	0.56	0.61	0.57
35	25.294	511-59-1	β-santalene	0.13	0.13	0.13	0.13	0.12	0.12	0.13
36	25.493	6753-98-6	α-Caryophyllene	0.11	0.11	0.09	0.12	0.12	0.12	0.12
37	25.871	6831-16-9	Aristolene	0.10	0.10	0.13	0.12	0.11	0.10	0.12
38	25.957	18794-84-8	β-farnesene	0.33	0.33	0.41	0.33	0.32	0.35	0.33
39	26.986	18252-44-3	cis-β-Copaene	0.60	0.60	0.71	0.61	0.61	0.60	0.61
40	27.265	28973-97-9	(6Z)-β-farnesene	0.12	0.12	0.10	0.13	0.12	0.12	0.13
41	27.594	5951-61-1	1,2,4a,5,8,8a-hexahydro-4,7-dimethyl-1-(1-methylethyl)-, (1R,4aS,8aR)-rel- Naphthalene	0.13	0.13	0.09	0.14	0.13	0.11	0.14
42	27.840	24703-35-3	3,6,6,9-tetramethyl-1,4,4a,5,7,9a-hexahydrobenzo [6]annulene	1.52	1.53	2.19	1.75	1.58	1.65	1.75
43	28.116	10208-80-7	α-Muurolene	0.08	0.09	0.07	0.09	0.08	0.08	0.09
44	28.483	489-29-2	β-Maaliene	0.12	0.12	0.12	0.13	0.12	0.10	0.13
45	28.673	495-61-4	β-Bisabolene	0.35	0.36	0.43	0.35	0.34	0.37	0.35
46	28.891	267665-20-3	1,2,4a,5,8,8a-hexahydro-4,7-dimethyl-1-(1-methylethyl)-, (1R,4aS,8aR)-rel- Naphthalene	0.17	0.17	0.50	0.17	0.19	0.39	0.17
47	29.008	13062-00-5	γ-bisabolene	0.26	0.28	0.29	0.26	0.26	0.27	0.26
48	29.406	483-76-1	δ-Cadinene	1.07	1.11	0.91	0.63	1.03	-	0.63
49	29.465	58319-04-3	(1S,5S)-1-[(2R)-6-methylhept-5-eSesquisabinen	-	-	-	0.42	-	0.96	0.42
50	29.925	53585-13-0	γ-Bisabolene	0.21	0.19	0.23	0.18	0.18	0.21	0.18
51	30.598	469-61-4	α-Cedrene	0.08	0.08	0.19	0.08	0.09	0.16	0.08
52	32.192	30021-74-0	γ-Muurolene	-	-	0.44	-	0.08	0.22	-
53	32.599	489-39-4	Aromadendrene	0.11	0.11	0.08	0.11	0.11	0.10	0.11
54	33.017	25246-27-9	Alloaromadendrene	0.09	0.08	-	0.08	0.08	0.04	0.08
55	33.717	21966-93-8	Copaborneol	0.10	0.09	0.07	0.08	0.09	0.08	0.08
56	34.571	3853-83-6	α-Himachalene	0.05	0.05	0.04	-	-	-	-
57	35.197	16728-99-7	1,4-Cadinadiene	0.08	0.08	-	0.07	0.07	-	0.07
58	35.998	5937-11-1	T-cadinol	0.17	0.17	-	0.15	0.16	0.10	0.15
59	36.606	17066-67-0	β-Selinene	0.13	0.13	-	-	-	-	-
60	36.707	133645-25-7	T-cadinol	0.25	0.25	-	0.23	0.37	0.22	0.23
61	37.615	178737-43-4	2,5-Methano-2H-1-benzopyran, octahydro-2,8a-dimethyl-6-(1-methylethyl)-, (2R,4aS,5S,6R,8aR)-rel-	0.11	0.11	0.12	0.09	0.11	0.11	0.09
62	37.757	145512-84-1	Sesquisabinene hydrate	0.12	0.11	0.11	0.10	0.11	0.11	0.10
63	38.072	19903-70-9	(E)-5-[(1S,3R,6R)-2,3-dimethyl-3-tricyclo[2.2.1.02,6]heptanyl]-2-methylpent-2-enal	0.07	0.12	0.08	0.06	0.07	0.07	0.06
64	38.553	23089-26-1	(-)-α-Bisabolol	0.13	0.13	0.15	0.12	0.13	0.13	0.12
65	38.691	515-69-5	α-Bisabolol	-	-	0.08	-	-	-	-
66	38.972	17699-05-7	α-Bergamotene	0.12	0.11	0.14	0.10	0.12	0.12	0.10
67	39.249	77-42-9	β-Santalenol	0.22	0.21	0.22	0.18	0.21	0.21	0.18
68	40.104	88034-74-6	α-Bergamotenol	0.23	0.23	0.24	0.20	0.23	0.24	0.20
69	46.777	115-71-9	(Z)- α-Santalol	0.08	0.08	0.11	0.08	0.08	0.09	0.08
70	47.550	698365-10-5	2-methyl-6-(4-methyl-1,4-cyclohexadien-1-yl)-, (2Z,6R)- 2-Hepten-1-ol	0.15	0.15	0.19	0.14	0.15	0.16	0.14

## Data Availability

The original contributions presented in this study are included in the article and Supplementary Materials. Further inquiries can be directed to the corresponding authors.

## Supplementary Materials

The following supporting information can be downloaded at: https://www.mdpi.com/article/10.3390/foods15081404/s1, Figure S1: The gas chromatogram of *A. villosum* samples obtained via seven drying methods; Figure S2: The standard curve of Camphor, Borneol and Bornyl acetate; Figure S3: The drying time of seven drying methods.
