# Supplementary material for: Effects of Different Drying Methods on the Quality of Amomum villosum Lour. Based on GC-MS and Chemometric Techniques
Source: Foods. 2026 Apr 17;15(8):1404. doi: 10.3390/foods15081404 (PMC13115150; doi:10.3390/foods15081404)
Supplement: Supplementary file 1 [file foods-15-01404-s001.zip › foods-4225402-supplementary.pdf]

Supplementary File

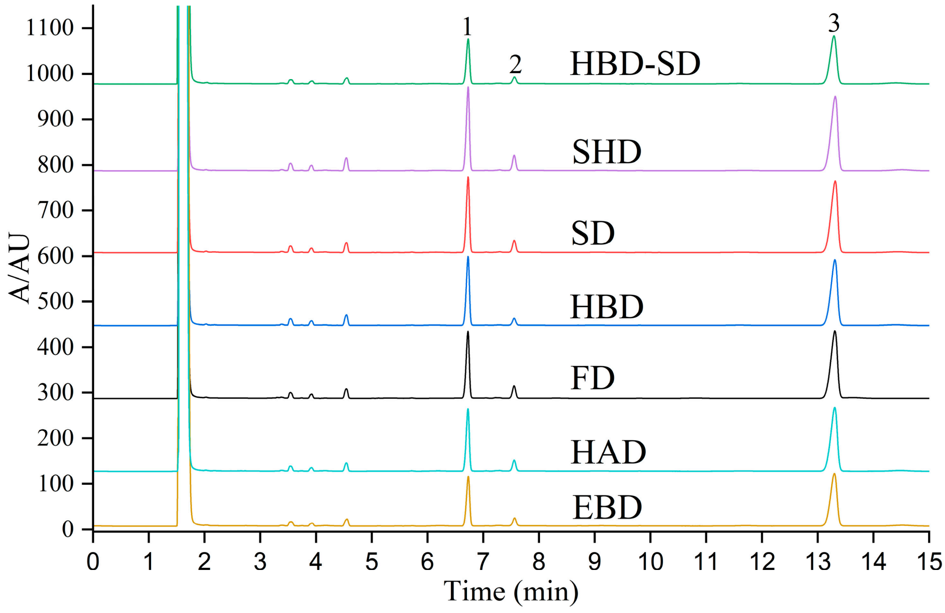

Supplementary Figure S1. The gas chromatogram of A. villosum samples obtained via seven drying methods. 1. Camphor 2. Borneol 3. Bornyl acetates.

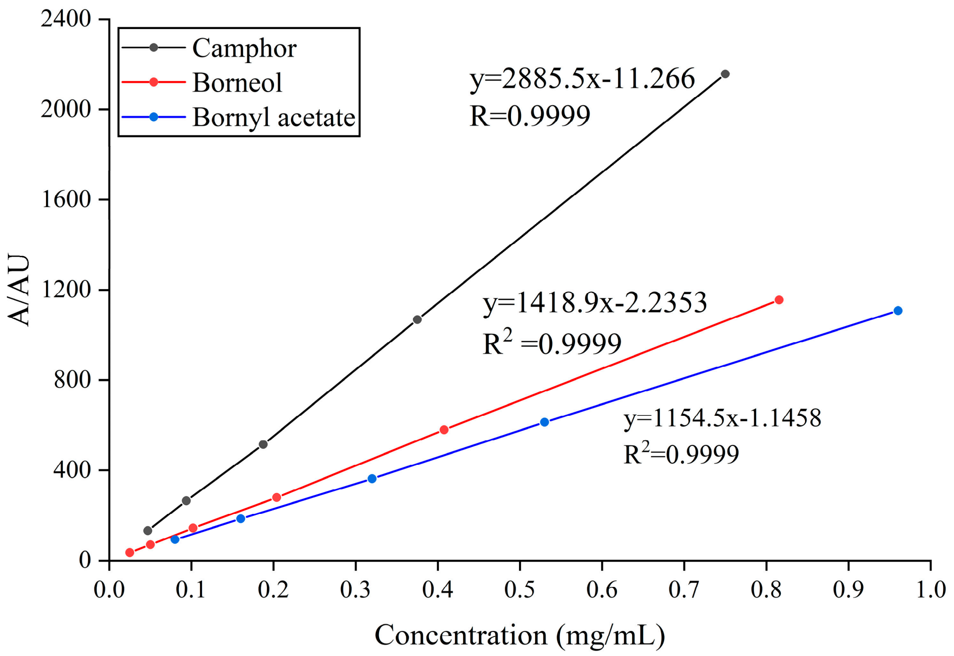

Supplementary Figure S2. The standard curve of Camphor, Borneol and Bornyl acetate

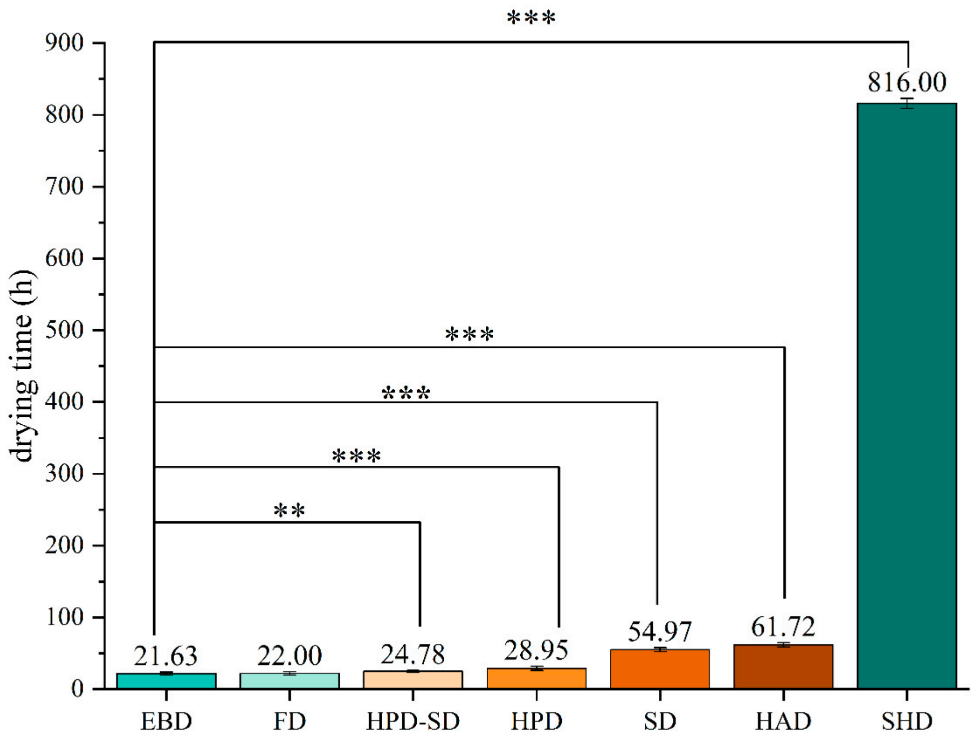

Supplementary Figure S3. The drying time of seven drying methods. Data are shown as means ± SD. The significant differences were calculated by the Student's t-test and expressed as an asterisk (* represents $p < 0.05$, ** represents $p < 0.01$, *** represents $p < 0.001$).
